# Halal Ethnomedicine as A Health Tourism Initiative: A Case Study from Bayan Village, Lombok

**DOI:** 10.21315/mjms-11-2024-878

**Published:** 2025-04-30

**Authors:** Taufiq Kurniawan, Ahmad Ahmad, Herman Supriadi, Hanofi Harianto

**Affiliations:** 1 Hungarian University of Agriculture and Life Sciences (MATE), Budapest, Hungary; 2 Universitas Negeri Manado, North Sulawesi, Indonesia; 3 Akademi Bisnis Lombok, West Nusa Tenggara, Indonesia; 4 Universitas Hamzanwadi, West Nusa Tenggara, Indonesia; 5 Universiti Sultan Zainal Abidin, Terengganu, Malaysia

**Keywords:** halal ethnomedicine, tourism product, marketing strategy, health tourism, case study, Indonesia

## Abstract

**Background:**

This study explores the integration of halal ethnomedicine (HE) into health tourism within Bayan Village, North Lombok, Indonesia. HE combines traditional medical practices with Islamic principles, offering a culturally resonant health care option. This research aims to assess the potential benefits and challenges of developing HE as a health tourism initiative.

**Methods:**

Utilising a qualitative case study approach, this research was conducted in Bayan Village, North Lombok Regency, West Nusa Tenggara Province, Indonesia. Data were collected through interviews with local stakeholders, including policy-makers, residents, and health tourists, from November 2023 to January 2024.

**Results:**

The findings indicate that the Bayan community strongly supports the development of HE-based health tourism, primarily because of its economic potential and cultural preservation. The financial impact of HE was highlighted as the most significant, with the community recognising its capacity to generate income and attract investment. However, the health benefits, while acknowledged, were not as strongly prioritised. This study also identifies several challenges, including low local interest, a financial focus that may hinder long-term development, the spread of misinformation, and the limited quality of local human resources. To address these challenges, this research proposes a collaborative model involving public and private sector partnerships. This includes strategies for community engagement, capacity building, and marketing, emphasising the importance of aligning HE with broader tourism and health care infrastructure.

**Conclusion:**

This study concludes that while HE holds promise as a health tourism product, its successful implementation requires overcoming significant sociocultural and operational barriers. This research contributes to the understanding of how traditional medicine can be integrated into modern health tourism, offering insights for policy-makers and stakeholders in similar contexts.

## Introduction

Health tourism has become increasingly significant in recent years. It encompasses a range of specialised categories, including thermal tourism, spa and wellness tourism, medical and dental tourism, senior tourism, and accessible tourism (1). Health tourism is seen as a means to advance society, resulting in more investment in various areas and generating employment, income, satisfaction, and vitality (2). Health tourism also contributes greatly to enhancing users’ happiness levels based on their individual profiles (3). Mathew (4) reported that health tourism has significant growth potential and contributes to the economic growth of countries such as India, as it has the ability to compete with developed nations in terms of infrastructure, technology, and expertise. Similarly, İldaş (5) noted that health tourism plays a crucial role in the promotion of a country and improving the economy of its population. When effectively managed, countries such as Turkey have the opportunity to become increasingly favoured by patients from around the world.

In the realm of health tourism, halal ethnomedicine (HE) has recently played a significant role, particularly for Muslim patients seeking culturally and religiously compliant health care services. HE pertains to the utilisation of medical plants and traditional remedies for the treatment of various ailments, encompassing halitosis and other health conditions. It constitutes a division of medical anthropology that scrutinises the significance of illness and examines the diverse convictions and customs associated with the human body, health, and malady in diverse cultures and communities. Ethnomedicine aspires to amalgamate cognition, conduct, and well-being by correlating mental disposition with physical welfare. It accentuates the harmony between curative medications and an individual’s beliefs, increasing placebo effects and fostering the recuperative process. Ethnomedicine also has potential as an antimicrobial botanical medicine for addressing halitosis (6). Nevertheless, Kintoko and Desmayanti (7) argue that the halal status of medication should be considered by authorities and health care providers to protect Muslim communities’ welfare and interests.

Thus, HE shows promise for advancement as a health tourism initiative. The exploration of halal medical tourism has promoted its progression, accentuating the increasing desire for halal inclinations and encouraging Muslim-friendly advancements and substantial commercial possibilities (8). Maripatul Uula and Ikhwan (9) stated that the global investigation of halal medical tourism has been undertaken, with notable contributions to research in this domain from countries such as Malaysia, Indonesia, the United States, South Korea, Australia, and China. However, Alfarajat (10) reported various obstacles and deficiencies in the delivery of halal food services within health care establishments, necessitating attention to augment the overall contentment of patients and their companions. Nurses in Indonesia acknowledge the notion of halal health care tourism and discern independence from its progress, underscoring the importance of effective leadership in ascertaining the primary areas of concentration within medical facilities (11). Overall, while HE in health tourism shows promise, addressing existing barriers is crucial for its sustainable development.

Bayan is a region characterised by rich cultural and religious practices, including HE. The community in this region utilises a diverse array of 36 plant species from 26 families for traditional medical purposes, addressing 32 different ailments through various preparation methods, such as boiling and grinding (12). Additionally, the local economy is shaped by regulations that align with sharia principles, aiming to foster sustainable development while preserving cultural heritage (13). However, challenges remain in fully empowering indigenous communities and ensuring that tourism development aligns with local knowledge and environmental sustainability (14).

This study posits that the amount of research conducted on HE, particularly on Lombok Island, remains low, as evidenced by the limited literature available. This dearth of research significantly constrains our comprehension of medical plant knowledge and practices in this region, as well as the potential for discovering novel medicinal plants and their applications (15). Consequently, a more comprehensive and focused study on HE is imperative, as it can contribute to governmental initiatives, particularly those in North Lombok. This research specifically endeavours to scrutinise HE as a nascent concept globally while simultaneously assessing its viability as a health tourism programme in the North Lombok area. Therefore, the aims of this research are as follows: i) to determine the benefits of HE as a health tourism programme; ii) to measure the impact of HE as a health tourism programme from the health, economic and social sectors; iii) to design a collaboration model between elements in managing tourism programmes involving halal ethnomedical-based health; iv) to determine the greatest challenges in realising a halal ethnomedical-based health tourism programme; and v) to design a model for implementing a halal ethnomedical-based health tourism programme in North Lombok Regency. From the problems and research objectives that have been stated, the following hypotheses are proposed:

H1: HE has positive effects on the welfare of society
H2a: HE has a positive effect on health
H2b: HE has a positive effect on local economic development
H2c: HE has a positive effect on the sociocultural situation of the local community

## Methods

This exploratory research utilised a qualitative approach employing a case study method. A case study is suitable for addressing intricate, context-dependent issues that require multiple perspectives and flexible designs (16). Thus, the case study was deemed the most appropriate choice for this research since, in accordance with Yin, the case study approach involves a comprehensive examination of a subject or occurrence within an authentic context (17). This research was conducted in Bayan Village, North Lombok, West Nusa Tenggara Province, Indonesia. North Lombok has been selected as a research site because: i) the longstanding practice of conventional ethnomedicine within the region has persisted for many years; ii) there is significant potential for traditional medicine to be conceptualised and marketed as HE; and iii) North Lombok holds the potential capacity to be developed as a health tourism destination predicated on the principles of HE.

This research applied purposive sampling to select the participants. Purposive sampling is a nonprobability sampling technique used to select informants with specific knowledge or characteristics relevant to a study (18). In this way, we segmented the interviewees into three distinct categories: policy-makers, local residents, and visitors seeking health care. Specifically, there were 50 participants, comprising 27 males and 23 females; the majority were aged between 30 and 70 years, as shown in Table 1. The rationale behind this decision was rooted in the assumption that these individuals would possess a wealth of life experience and practical knowledge pertaining to the utilisation of HE for health-related purposes. Furthermore, they also possessed a comprehensive understanding of HE itself, coming from villages outside of the selected area but frequenting the location for medicinal purposes, and were permanent residents of the villages in which the research was conducted.

### Data Collection

In this study, data collection was carried out in both primary and secondary manners. Primary data were obtained through direct observation and in-depth interviews. We performed data collection from November 2023 to January 2024. First, the collection of research data was executed through direct observation techniques within the villages. This method was implemented on five occasions, resulting in the creation of various field notes that included photographic evidence, research documentation, as well as face-to-face interviews with the participants. Moreover, secondary data were obtained through theoretical studies obtained from various sources, such as documentation from several village monographs, Regional Owned Enterprises of West Nusa Tenggara Province reports, and literature studies in scientific journals. Specifically, these secondary data include censuses, government records, and organisational data. While using existing documents may seem straightforward, we were also aware of methodological challenges such as document selection, availability, and potential biases in the original data collection.

### Data Analysis

**Figure 1:**
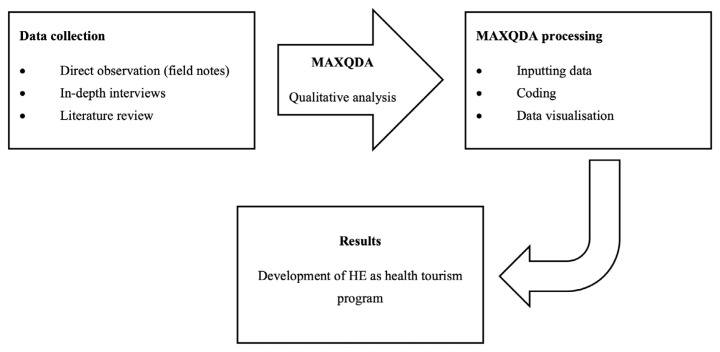
Research flow chart

The data were analysed using MAXQDA software. The data analysis process using MAXQDA involves several systematic steps (19) and serves as a versatile tool for enhancing the rigour and efficiency of data analysis (20). Initially, the data collected from interviews were transferred to a computer device and transcribed word-for-word. The transcription results were grouped into relevant themes, adjusting the needs of the findings for this article. The theme grouping process began during the field data collection process, and only themes relevant to this study’s purpose were eventually selected. The data processing was then carried out by inputting data, coding data based on themes, generating visualisations, and presenting the data descriptively. All of these processes are illustrated in Figure 1, which was adopted from a previous study conducted by Soeswoyo and Dewantara (21).

## Results

### Profile of Bayan Village

Bayan is a subdistrict located in North Lombok Regency, West Nusa Tenggara Province, and is also the subdistrict with the largest number of villages in the regency. The majority of people in the Bayan area are Sasak people who follow the Islamic religion. The name Bayan itself is the name of the village as well as the name of the subdistrict. This area was named by a saint who spread Islam in the area so that Bayan would later become a light for other areas on the island of Lombok. Bayan Village is approximately five kilometres from the beach, is located on the slopes of the Rinjani Mountains, and is at an altitude of 200 metres above sea level. It is also in this area that the heartbeat of ancient culture can always be heard, a culture that is far outside the dynamics of life and that manifests itself in various other places on the island of Lombok. This ancient and traditional culture still survives and provides a unique and particular style to the Islamic religion.

There are nine hamlets in Bayan Village itself: East Bayan, West Bayan, Padamangko, Mandala, Montong Baru, Teres Genit, Tutul Hamlet, and Nangka Rempek. Among these hamlets, the hamlets of East Bayan (Timuq Orong), West Bayan (Bat Orong), Karang Salah, and Karang Bajo are considered the centre of Bayan because all political activities, including social, religious and sociocultural activities, are held in these four hamlets.

The data in Table 2 indicate that Bayan District had the largest population in 2020, reaching 44,671 people. The subdistrict with the lowest population was Selamat District, with a population of 32,546 people.

### HE in Bayan: History and Concept

This research examines the beliefs and practices of the Sasak Muslim community in North Lombok Regency. It combines traditional cultural development with modern health concepts and gives attention to medical anthropology issues. This framework introduces a new definition that combines “halal” and “Ethnomedicine” in relation to the people of North Lombok. Research was conducted on the ethnomedicine of the Sasak community in Bayan Village, North Lombok Regency, in 2009. The findings were tested at the Faculty of Public Health, Hasanuddin University. The examiners noted that certain ethnomedicine practices conflict with Islamic teaching. These practices involved the cultural rituals of the Sasak people, which went against Islamic values, followed by the majority of the Sasak community. The examiners cautioned the writers about assessing cultural differences due to the sensitivity of the topic. The rituals included traditional alcohol consumption (the so-called *berem*/*beloq*) and the use of evil jinn to make someone sick. The field of ethnomedicine in North Lombok has a long history, but Islamic cultural values are more prevalent in society.

The researchers posit that the disparities in halal-haram cultural values do not pose a divisive threat to society but rather represent a reservoir of Sasak cultural values that can be harnessed as a vast potential for the tourism sector in North Lombok. Broadly speaking, the utilisation of “halal” extends beyond the confines of the Muslim community, as it ensures the safety and comfort of all segments of society. The halal principle is equitable for all groups, as it enables all members of society to partake in and avail themselves of products and services that adhere to halal standards. Conversely, predicaments may arise in the absence of established benchmarks for a given product or service. To illustrate, if a product or service lacks halal certification, then the Muslim populace is precluded from enjoying and analysing that product and service. The concept of “halal” is not exclusively limited to Muslim consumers, despite its primary focus being on this demographic.

### Advantages of HE

#### Local Perspectives Regarding the General Opportunities Most Likely Generated from HE

**Figure 2:**
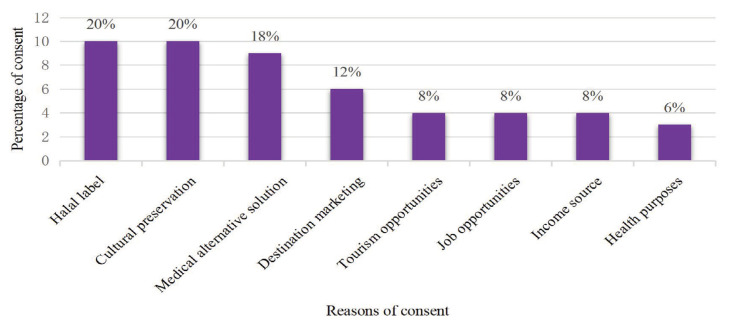
Local society perspectives on HE-based health tourism programme in Bayan Village

The practice of HE in Bayan Village is a culture that has developed for a long time, still survives today, and has been commonly carried out by most people in Bayan Village for generations. According to several findings, the sustainability of this practice is due to the rich biodiversity in Bayan, which supports the use of numerous plant species that are integral to local healing practices (12), and the traditional knowledge that has passed through generations, ensuring the continuity of ethnomedicine (22, 23). Next, we tested the sustainability of ethnomedicine practices by implementing a halal label as a product in the development of health tourism in Bayan Village. In this case, we asked for local community responses regarding their reasons for agreeing to the development of HE practices and its projection as a health tourism programme, as shown in Figure 2.

The responses shown in Figure 2 indicate that, in general, there were eight reasons given by the Bayan community for developing health tourism: prioritising HE practices as the main product as illustrated in Figure 1, “halal label” and “cultural preservation,” which both with 20% of votes, are the strongest reasons why the public supports this project. “Medical alternative solutions” and “destination marketing” were the next reasons, with 18% and 12% of votes, respectively. Moreover, “health purpose” was actually the reason for the lowest number of votes, namely, 6%.

#### Financial, Health, and Social Impacts Generated by HE

**Figure 3:**
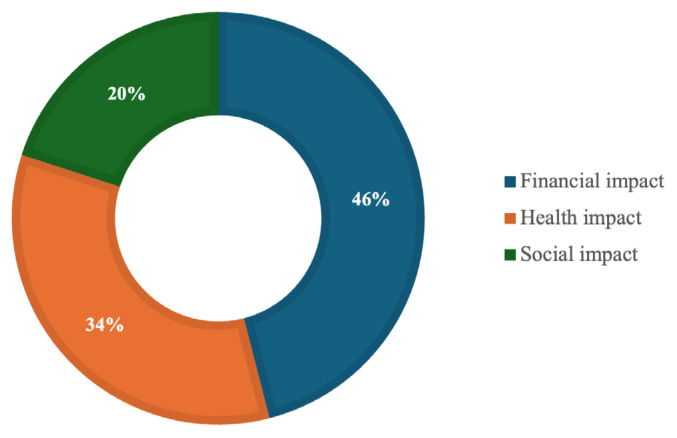
Financial, health, and social impacts of HE

Furthermore, we specifically tested three important aspects that are benchmarks for the development of HE-based health tourism in Bayan Village, namely, health, financial and social. In this case, we analyse how much HE, as a health tourism programme, has positive effects on these three aspects, as shown in Figure 3.

Based on the analysis carried out from the interview results in Figure 3, it can be concluded that the financial aspect has the greatest impact on the HE programme, with 46%. On the other hand, surprisingly, the positive impact of HE programmes on health is not as great as the financial impact, with only 34%. Moreover, the social aspect has the least impact, with a contribution compared with the previous two aspects with 20%.

### Collaborative Model for Developing HE as a Health Tourism Destination in Bayan Village

In this section, we analyse what must be done by stakeholders involved in realising the HE-based health tourism programme in Bayan Village. In this case, we compile and conclude all the responses from the respondents in table form (see Table 3). For this analysis, we specifically examined responses from only a few respondents who were qualified to answer questions related to this development model. The respondents we chose were as follows: i) local government policy-makers, marked with code R1; ii) head of Bayan Village, marked with code R2; and iii) head of Tourism Awareness Group, marked with code R3.

The results of this analysis in Table 3 indicate that five indicators serve as references for mapping the HE-based halal tourism programme development model: socialisation, community involvement, community empowerment, traditional school provision, and social media promotion.

### Challenges to Address Prior to the HE Programme’s Realisation

On the other hand, we also analysed the greatest challenges found in the realisation of the HE-based health tourism programme in Bayan Village. In this case, we carry out the same pattern as the previous pattern in terms of matching the question with the respondent’s ability to respond (see Table 4). Therefore, for the questions related to this challenge, we chose the Village Head (R2) and the Head of Tourism Awareness Group (R3) as responders.

An analysis of the results of the interviews in Table 4 revealed four indicators that were challenges in developing a HE-based health tourism programme in Bayan Village. The four indicators are the lack of local interest (local interest), community finances that are too salary-oriented without prioritising the process (financial orientation), the frequent spread of hoax news related to the negative impact of HE (hoax info), and the less optimal quality of local human resources available (local human resources quality).

### HE Marketing Model

An equally important part is the marketing pattern of HE as a product of health tourism. In this case, we analyse how stakeholders relate to what type of marketing strategy will be used for HE products so that they can be marketed optimally (see Table 5). According to the data we obtained from interviews with the Head of Tourism Awareness Group (R3), five steps are taken, as shown in Table 5: i) collaboration with the local health department to guarantee the quality of products and services; ii) collaboration with several local hotels and clinics; iii) becoming the main producer and supplier of HE, both products and services; iv) involving the local government as a promoter; and v) procurement collaboration with several investors by the local government.

**Figure 4:**
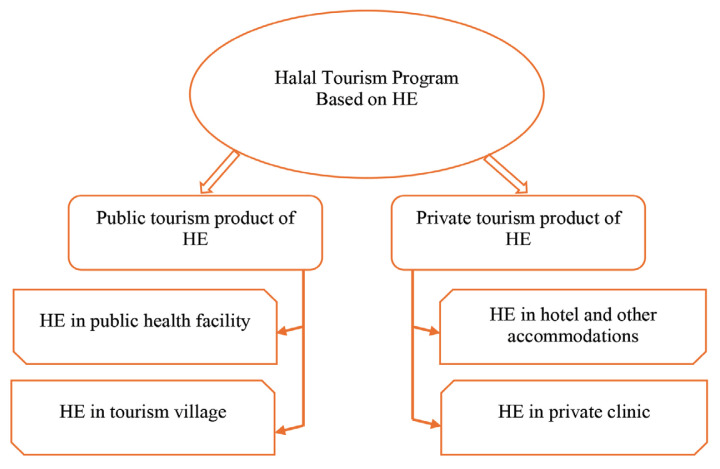
Distribution of HE products in the public and private sectors within local tourist spots

As a comprehensive conclusion, we designed an HE development framework and marketing strategy, as shown in Figure 4. In this case, development and marketing are distributed to two different sectors, namely, the public sector (public tourism product of HE) and the private sector (private tourism product of HE). The public tourism products of HE include public health facilities such as regional general hospitals and tourism villages such as Bayan Village. HE’s private tourism products include hotels, accommodations, and private clinics.

## Discussion

The findings from this research offer a nuanced understanding of the cultural and socioeconomic dynamics within Bayan Village, particularly in the context of the practice and potential development of HE as a health tourism product. The insights reveal both the opportunities and challenges associated with integrating traditional Sasak practices with contemporary health tourism initiatives, particularly within the framework of Islamic values.

### Cultural Significance and Continuity of HE

The practice of HE in Bayan Village is deeply rooted in the region’s rich cultural heritage and biodiversity, which has supported traditional healing for generations. The continued use and preservation of these practices are indicative of a strong cultural identity among the Sasak people, who have managed to harmonise their ancient traditions with Islamic principles. This coexistence of traditional culture and religion is unique to Bayan, making it an alternative proposition for cultural and health tourism. The community’s support for this initiative, as evidenced by the prioritisation of the “halal label” and “cultural preservation,” underscores the importance they place on maintaining their cultural heritage while embracing new economic opportunities.

The cultural significance and continuity of ethnomedicine as a health tourism programme are closely intertwined with the sociocultural dynamics of the communities involved. Ethnomedicine is not just a health care option; it also embodies the cultural heritage and identity of indigenous populations, making it a vital component of health tourism. Numerous studies have highlighted the importance of ethnomedicine, emphasising that it reflects indigenous healing practices rooted in cultural traditions, as seen in communities such as the Tharu of Nepal (24). These practices offer essential health care options for local populations while preserving cultural continuity. The integration of cultural values into health practices enhances the appeal of health tourism, as tourists seek authentic experiences that connect them to local traditions (25). Moreover, ethnocultural preparation for medical tourists is crucial, as it fosters understanding and respect for local practices and enriches the overall experience (26). Promoting ethnomedicine within medical tourism not only attracts domestic and international tourists but also contributes to the sustainability and preservation of these cultural practices (25).

### Socioeconomic Impacts of HE-based Health Tourism

In terms of socioeconomic impacts, the findings highlight the substantial financial impact that the development of HE-based health tourism could have on the Bayan community. The community’s recognition of financial benefits, as indicated by 46% support for this aspect, suggests a strong economic incentive to pursue this initiative. This economic potential is further bolstered by the integration of HE with the broader tourism industry, particularly through destination marketing strategies that position Bayan as a unique cultural and health destination.

The socioeconomic impacts of ethnomedicine on health tourism programmes are multifaceted and significantly influence both local economies and cultural dynamics. By integrating traditional healing practices, ethnomedicine enhances the appeal of health tourism destinations, driving economic growth and fostering cultural exchange. Several studies have highlighted the socioeconomic benefits of developing ethnomedicine as a part of health tourism initiatives. For example, Ramanauskas (27) noted that health tourism can substantially boost local economies through job creation and increased investment in health care infrastructure. Similarly, Eusébio et al. (28) reported that social health tourism programmes, particularly those aimed at seniors, have high multiplier effects, generating significant economic benefits, including increased household income and employment opportunities. However, the sociocultural impacts of health tourism can also lead to disparities in health care access for local populations, underscoring the need for effective regulatory frameworks to ensure equitable service distribution (29). Without proper management, the influx of medical tourists may strain local health care systems, particularly affecting low-income groups (27).

Interestingly, the health impact, while significant, was not as highly prioritised by the community (34%) compared with the financial benefits. This suggests that while the health benefits of HE are recognised, they may not be the primary driver of community support. Instead, the emphasis appears to be on the economic opportunities that HE-based health tourism could bring to the region. The relatively lower emphasis on social impacts (20%) could indicate a need for further community engagement and education on the broader societal benefits of such an initiative.

### Challenges in Realising HE-based Health Tourism

Despite their promising potential, these findings also identify several challenges that need to be addressed to develop HE-based health tourism in Bayan successfully. The lack of local interest, the financial orientation of the community, and the spread of misinformation pose significant barriers to the programme’s realisation. Additionally, the quality of local human resources was identified as a critical challenge, suggesting a need for capacity building and education to ensure the success of this initiative.

Addressing these challenges will require a coordinated effort among stakeholders, including local governments, community leaders, and the tourism industry. This research highlights the importance of socialisation, community involvement, and empowerment as key strategies for overcoming these obstacles. Moreover, the role of traditional schools and social media promotion is highlighted as crucial in raising awareness and fostering community support.

Previous studies have highlighted the integration of ethnomedicine into health tourism programmes as a promising yet challenging endeavour. These challenges arise from cultural, regulatory, and operational factors that impact both practitioners and tourists. Wanzala and Minyoso (30) noted that ethnomedicine often faces polarised views, ranging from scepticism to idealisation, which can influence its acceptance in health tourism settings. Chandra et al. (31) emphasised the lack of standardised practices and regulations governing ethnomedicine, raising concerns about its safety and efficacy. Additionally, medical tourists may struggle to adapt to the ethnocultural nuances of the health care environment, potentially affecting their overall experience (32). Furthermore, the limited sociological research on ethnomedicine within the context of medical tourism restricts a comprehensive understanding of its potential benefits and challenges (32).

### Collaborative Development and Marketing Strategies

The development of HE-based health tourism in Bayan Village will necessitate collaboration between the public and private sectors. The proposed development model emphasises the need for partnerships with local health departments, hotels, clinics, and investors to ensure the quality and marketability of HE products and services. The dual approach of promoting both public and private tourism products reflects a comprehensive strategy that leverages Bayan’s unique cultural assets while addressing the diverse needs of different market segments.

The involvement of local governments as promoters and facilitators of these initiatives is particularly important in ensuring that development aligns with both cultural preservation and economic growth objectives. These findings suggest that a well-coordinated marketing strategy involving collaboration across various sectors is essential for positioning Bayan as a leading destination for halal health tourism.

The integration of ethnomedicine into health tourism programmes offers a unique opportunity to enhance collaborative development and marketing strategies. By leveraging the rich cultural heritage associated with traditional medical practices, this approach can attract both domestic and international tourists. Previous studies have explored how ethnomedicine can be marketed as a key component of health tourism programmes. Dewi and Ayuningtyas (33) argued that effective marketing strategies that incorporate ethnocultural elements can increase competitiveness among health care providers, particularly through targeted segmentation and positioning, to attract tourists seeking medical services. Additionally, Ural et al. (34) suggested that traditional practices have long attracted travellers, indicating that modern marketing efforts can build on this legacy. Despite the promising potential of ethnomedicine in health tourism, challenges remain in standardising practices and ensuring safety. Further research is needed to evaluate the efficacy of these traditional methods and their integration into contemporary health care frameworks.

In conclusion, the development of HE-based tourism in Bayan Village presents a significant opportunity to leverage the region’s cultural heritage and biodiversity for economic gain while preserving traditional practices. However, realising this potential will require addressing several challenges, including community engagement, misinformation, and capacity building. The findings provide a roadmap for stakeholders to develop collaboratively and market HE-based tourism, ensuring that the initiative not only benefits the local economy but also respects and preserves the unique cultural identity of the Sasak people in Bayan.

## Conclusion

The development of HE-based health tourism in Bayan Village represents a promising opportunity to harness the region’s rich cultural heritage and biodiversity for economic advancement while preserving traditional Sasak practices. The research findings indicate that the community recognised the substantial financial benefits that could arise from this initiative, with strong support for integrating the “halal label” and promoting cultural preservation as key drivers. Despite the potential economic gains, the community places relatively less emphasis on the health and social impacts of HE, highlighting a need for increased awareness and education on the broader benefits of the programme.

Moreover, the successful implementation of HE-based tourism will require overcoming significant challenges, including local disinterest, financial orientation, misinformation, and the limited capacity of local human resources. Addressing these issues necessitates a coordinated effort among stakeholders, emphasising community involvement, empowerment, and strategic partnerships with both the public and private sectors. By fostering collaboration and implementing targeted marketing strategies, Bayan Village can position itself as a leading destination for halal health tourism, benefiting both the local economy and the preservation of Sasak’s cultural identity.

This study has several limitations that should be considered when interpreting the findings. First, the research is geographically limited to Bayan Village, which may not fully represent the broader Sasak community or other regions with similar ethnomedical practices, and it may not capture the full diversity of perspectives within the community. Additionally, the focus on HE may overlook other significant cultural practices and beliefs that are integral to the region’s health tourism potential. Finally, while this study provides valuable insights into the socioeconomic impacts and challenges of HE-based health tourism, further research is needed to quantify these impacts and evaluate the long-term sustainability and efficacy of integrating traditional practices into contemporary health tourism frameworks.

## Figures and Tables

**Table 1:** Demographic features of participants in the study area.

Demographic features		*n* = 50	Percentage (%)
Gender	Male	27	54
	Female	23	46

Age group	30–40	8	16
	41–60	21	42
	61–70	16	32
	> 71	5	10

Target group	Policy-makers	1	2
	Local residents	10	20
	Visitors	39	78

Main occupation	Local government	1	2
	Teachers	9	18
	Housewife	17	34
	Civil servant	3	6
	Retired	5	10
	Casual worker	4	8
	Entrepreneur	5	10
	Jobless	6	12

**Table 2:** Total population broken down by sub-district in North Lombok Regency in 2020

Subdistricts	Males	Females	Population (people)
Pemenang	16,462	16,084	32,546
Tanjung	21,962	22,644	44,606
Gangga	19,966	20,870	40,836
Kayangan	18,334	19,079	37,413
Bayan	21,943	22,728	44,671

Total	98,667	101,405	200,072

**Table 3:** Respondents’ responses on the collaborative model of HE development as a health tourism programme

Respondents	Comments	Indicators
R1	… the Village Head will convey this socialisation to village officials, staff and the community in general so that what we socialise is accepted as a whole. For the local community itself, we want to provide outreach about the importance of this medical practice as a health tourism programme. Because this will have a financial impact on the community itself, as well as an opportunity to introduce the village to the eyes of the world.	Socialisation
	We will also ask the local community there to want to be empowered and get involved as drivers of development. Because after all, this programme comes from the community, by the community, and for the community itself.	Community empowerment and community involvement
R2	… carry out outreach to the community itself as a form of our approach regarding the importance of utilising the potential that the village has, namely Halal-based traditional herbal medicine, to be used as a health tourism programme. We will socialise the positive impact or influence on society if this programme can be developed.	Socialisation
	… we must involve the community in every process and policy taken in developing this programme. Because as we know, the community will be more enthusiastic if they are also given a share in participating in building this programme.	Community involvement
R3	… we from Pokdarwis (Tourism Awareness Group) and village officials are working together to create a traditional school. The aim is for us to make it a place of learning for young people and the local community.	Traditional school provision
	… through this traditional school, we aim to empower youth and the community regarding the importance of cultural preservation and tourism development practices through existing potential.	Local empowerment
	Apart from that, as I said before, we from the Pokdarwis (Tourism Awareness Group) and village officials will work together to carry out large-scale promotions. Where we will go through social media …	Social media promotion

**Table 4:** Respondents’ responses on challenges towards HE-based health tourism programme realisation

Respondents	Comments	Indicators
R2	One of the big challenges faced in the realisation of this programme is how to increase the interest of our own community and maintain that interest. Because developing potential certainly requires time and a long process.	Local interest
	… most of our society today wants instant things. This means that when they work, they have to get money or wages. Well, this is what I think is the biggest challenge for me as Village Head, namely increasing the community’s interest in getting involved in developing the health tourism programme through the potential that we have.	Financial orientation
R3	… there is bad information, whether from people who do not support it or other individuals, which appear to be aimed at bringing down plans like this. Because one of the negative cultures that still exists here is the attitude of opposition to change by a handful of people. So, of course, this will have an impact on the development process of the programme that will be realised.	Hoax info
	… the quality of our human resources is still not optimal. In my opinion, the quality of human resources is very important in efforts to build the potential that exists in our villages.	Local human resources quality

**Table 5:** Respondents’ responses on the HE marketing model

Respondents	Comments	Indicators
R3	… we will collaborate with people who have fashion and expertise in the local health department.	Collaboration
	… from the tourism side, we from Pokdarwis (Tourism Awareness Group) will also collaborate with some local hotels and clinics.	
	We want this product only to be bought and sold at our place, aka the manufacturer and supplier only from us. I think this is a good thing to do to reduce competitors and keep the community focused on helping its development, and we are currently planning that.	Main producer and supplier
	… we will also collaborate with the village and regional government to become involved as promoters.	Collaboration
	The regional government, itself reportedly, will try to find investors as a first step in developing this programme.	Finding investors
